# Mechanisms of change in an online acceptance and commitment therapy intervention for insomnia

**DOI:** 10.1038/s41598-025-87018-3

**Published:** 2025-01-22

**Authors:** Tetta Hämäläinen, Päivi Lappalainen, Sitwat Usman Langrial, Raimo Lappalainen, Noona Kiuru

**Affiliations:** 1 Department of Psychology, University of Jyväskylä, P.O. Box 35, Jyväskylä, 40014 Finland; 2 Department of Behavioral Sciences, S3H, National University of Science and Technology, Islamabad, Pakistan

**Keywords:** Acceptance and commitment therapy, Insomnia, Online intervention, Mechanisms of change, Thought suppression, Psychology, Human behaviour

## Abstract

Insomnia, i.e., difficulty falling asleep or staying asleep, is a common condition that is connected to many psychological and physical problems. Online-based Acceptance and Commitment Therapy (ACT) has recently been introduced as an option for treating insomnia. However, our understanding is yet limited on what happens during an online ACT intervention or what underlying mechanisms are critical for outcomes. This study addressed this gap by investigating mediators of a brief self-guided online ACT intervention for adults suffering from subclinical and clinical insomnia. A total of 86 adults were randomized to an intervention group (*n* = 43) or a waitlist control group (*n* = 43). Mediator models were used to investigate the effects of online ACT on subjective sleep complaints through changes in daytime sleepiness, dysfunctional beliefs, psychological symptoms, mindfulness, and thought suppression. Two models showed significant indirect effects: The online ACT intervention decreased participants’ thought suppression and depressive symptoms, which then decreased subjective sleep complaints. Other models did not yield significant mediating effects. Acceptance and mindfulness-based approaches may serve as viable options for other existing insomnia treatments. Future studies are encouraged to be conducted, especially concerning flexibility and inflexibility processes as possible mechanisms of change in online ACT for insomnia.

## Introduction

Insomnia is a common sleep disorder where a person experiences difficulties with falling asleep, maintaining sleep, or waking up too early^1^. Around a third of adults report some insomnia symptoms at any given moment, and 6–10% have symptoms that meet the criteria for a disorder^1,2^. Chronic insomnia exposes individuals to various health-related consequences such as increased risk of hypertension, diabetes, heart attack, depression, anxiety, and suicide^3^. The consequences of insomnia and sleep complaints are also significant for society, as they are connected to increased work absenteeism, sick leave, lower productivity, higher risks of accidents, and increased usage of healthcare services^4^.

### Interventions for insomnia

Cognitive behavior therapy for insomnia (CBT-I) is a widely recommended and effective treatment for primary and comorbid insomnia^5,6^, in face-to-face as well as in digital forms^7,8^. However, CBT-I comes with some limitations. First, noncompletion rates for individual and group-based CBT-I have ranged from around 10–39%^9^ and around 15% (ranging from 0 to 44%) for self-help CBT-I^10^. Some of the behavioral elements, such as restricting time in bed, have been regarded as difficult to adhere to, possibly due to higher fatigue at the early stages of treatment^11,12^. It has been suggested that restricting sleep may feel counterintuitive to patients trying to sleep more and even present risks for those whose work involves, for example, driving or operating dangerous machinery^13,14^. Second, focusing on controlling insomnia symptoms may increase cognitive-emotional arousal, which, in turn, may prolong falling asleep^15,16^. Attempting to control symptoms may thus work to the benefit of insomnia rather than alleviate it.

Methods using acceptance and mindfulness-based strategies have been suggested as one way of working with unhelpful cognitions and around the relationship one has with insomnia^17,18^. Acceptance and commitment therapy (ACT^15^) represents a third-wave behavior therapy that aims to increase flexibility in one’s responses to personal experiences and find ways to live a personally meaningful life. ACT sees psychological suffering as the cause of attempting to control or avoid cognitions and experiences that cause discomfort^15^. Thus, instead of trying to change or control the content of cognitions or personal experiences, attention can be targeted towards their functions and contexts^12^.

ACT aims to increase psychological flexibility, which consists of six psychological processes that are acceptance, cognitive defusion, mindfulness, self-as-context, values, and values-based actions^15^. An ACT intervention for insomnia would incorporate these in the following manner: First, instead of “fighting” with insomnia and trying to control sensations related to sleeplessness, ACT can be used to foster acceptance towards uncomfortable experiences^11,19^. Second, learning to recognize thoughts and emotions for what they are and create space for them rather than struggling with them or seeing them as something that needs to be eliminated; in ACT, this is referred to as cognitive defusion^15^. Third, practicing mindfulness increases awareness of the present moment and reduces sleep-related attentional bias^20^. Fourth, increasing one’s awareness of the flow of sleep-related experiences without evaluations of them or identifying with them, referred to as self-as-context^19^. Fifth, clarifying personal values or things that are important and improve the quality of life. In an ACT intervention for insomnia, values clarification is done while refocusing attention away from sleep rather than around controlling sleep^11,20^. Lastly, ACT can be used to promote commitment to actions that are in line with one’s values and develop goals that are pursued regardless of insomnia^12,19^.

Several studies have demonstrated ACT interventions to be effective in improving adult sleep quality and insomnia symptoms compared to waitlist control (e.g., see reviews by^18,21,22^). Lately, also online-based ACT interventions have been introduced for primary and comorbid insomnia^23,24^. Comparisons between ACT and CBT-I have suggested both to be effective intervention forms^25–27^, although CBT-I has demonstrated larger effect sizes^22^. However, ACT has been associated with better participant adherence^28^ and has been suggested to fit especially those who do not respond to CBT-I or who have high insomnia-related anxiety^27^. In their meta-analysis comparing ACT and CBT across various health conditions, Fang and Ding^29^ suggested that ACT may be more effective for individuals with behavioral problems and improving mindfulness skills, whereas CBT may be more effective in treating physiological symptoms and anxiety.

### Mechanisms of change in ACT interventions

Investigating mediators and mechanisms of therapeutic change is important to understand what is critical to treatment and help the research be shifted to real-world settings^30^. While there is evidence of the benefits of mindfulness and acceptance-based approaches for improving the symptoms of insomnia, more research is needed to investigate which mechanisms lie behind changes^17,22,31^. To our knowledge, previous mediational RCT studies have not been conducted with online ACT for insomnia, but studies using other outcome measures can be found. Trompetter et al.^32^ found psychological flexibility and pain catastrophizing to mediate the effect of online ACT on chronic pain, whereas Pots et al.^33^ found psychological flexibility and mindfulness to mediate the effect of online ACT on depression. Practices on non-reactivity mediated the effect of a blended ACT intervention on university students’ stress, depression, and well-being^34^, and changes in depressive symptoms mediated the effect of online ACT on adolescents’ subsequent school engagement^35^. In their systematic review of mediators of face-to-face and online ACT interventions, Stockton et al.^36^ found that acceptance was a mechanism of change regarding mental health and patient functioning and, to a limited extent, regarding the quality of life and behavioral outcomes.

In their metacognitive model, Ong et al.^17^ proposed that increasing awareness of the presence of insomnia-related symptoms facilitates a shift in one’s relationship with them. That is, instead of attempting to change a thought, the acceptance and mindfulness approach promotes greater awareness of sleep-related distressing thoughts and adopts a non-judging and accepting stance toward them^17^. Similarly, Lundh^37^ suggested that interventions focusing on acceptance and mindfulness may reduce symptoms and promote quality of sleep because insomnia is perpetuated by sleep-interfering and dysfunctional sleep-interpreting processes. Dalrymple et al.^11^ integrated ACT principles with CBT-I-based behavioral techniques and suggested that especially letting go of efforts to control sleep and accepting short-term discomfort produced by sleep restriction may be related to commitment to behavioral strategies.

### Thought suppression and insomnia

Thought suppression refers to the cognitive strategy of attempting to control and eliminate the occurrence and impact of unwanted thoughts, behavioral patterns, or emotional experiences^38,39^. It can be regarded as a form of experiential avoidance, which refers to the unwillingness to remain in contact with private experiences^40,41^. Experiential avoidance is part of a broader construct of psychological inflexibility. Psychological inflexibility (in contrast to flexibility) is a transdiagnostic pathological process associated with psychological problems and disorders, including insomnia^15,31,42,43^. From an ACT perspective, working with avoidance of unwanted or unpleasant sleep-related sensations would require increasing awareness of them and willingness to make room for and experience them.

Suppression of emotionally distressing thoughts may lead to increased emotional reactions and arousal of the sympathetic nervous system^44^, which can have a negative impact on sleep. Indeed, sleep complaints and a higher frequency of unwanted thoughts have been associated with the usage of unworkable cognitive strategies such as suppression^45–47^. People suffering from insomnia seem more likely to utilize suppression compared to good sleepers, although the adverse effects of suppression on sleep quality and sleep onset can be observed in both^48^. In contrast, a better quality of sleep has been associated with strategies such as social control (i.e., whether one shares their thoughts with others) and replacement^45^, and lower distress caused by intrusive thoughts has been attributed to the usage of acceptance and interesting or engaging distractions^49,50^. In addition, thought suppression has been found to mediate the associations between negative mood and post-traumatic stress^51^, trauma exposure and post-traumatic stress^52^, and depressed mood and opioid cravings^53^. Overall, previous literature seems to support the view that thought suppression is a cognitive strategy associated with psychopathology and that interventions that target insomnia should address thought suppression as one central process in it.

### Aim of the present study

Few experimental studies have investigated the effects of ACT on primary and comorbid insomnia. Lappalainen et al.^54^ found that the participants receiving an online ACT intervention experienced improvements in their sleep quality, sleep duration, dysfunctional cognitions, thought suppression, and depressive symptoms, with effects maintained at a 6-month follow-up. Studies by Rickardsson et al.^23,24^ reported an online ACT intervention for chronic pain to improve pain interference, as well as insomnia and other comorbid symptoms. Another study investigating a group-based ACT intervention for clinical insomnia discovered that decreased experiential avoidance was associated with improvements in participants’ sleep complaints^55^. Recent systematic reviews, although mostly addressing studies investigating face-to-face ACT interventions, have underlined ACT as a viable option for treating insomnia^18,21,22^.

However, little is known about the underlying mechanisms of change in an online ACT intervention for people with sleep complaints^23,24^. The present study aimed to address this gap by examining the indirect effects of a six-week online ACT intervention on subjective sleep complaints in adults experiencing clinical or sub-clinical insomnia. Sleep complaints, i.e., the main outcome, were operationalized in this study using the Basic Nordic Sleep Questionnaire (BNSQ^56^), which is an assessment tool that includes a wide range of sleep-related health complaints^57^. The present study investigated changes in daytime sleepiness, beliefs about sleep, mindfulness, thought suppression, overall psychological distress, and depressive symptoms as possible mediators from an online ACT intervention to subjective sleep complaints.

## Methods

### Participants and procedure

Data collection was conducted between October and November 2013. The study was carried out following the Code of Ethics of the World Medical Association (Declaration of Helsinki). The study was approved by the Ethical Committee of the University of Jyväskylä. The RCT consort guidelines were followed throughout the study; however, the trial was not registered in the ClinicalTrials.gov Protocol Registration.

Participants were recruited by placing advertisements in local newspapers (cities of Jyväskylä and Oulu, Finland) stating that we were looking for volunteers suffering from sleep difficulties. The following criteria were set for eligibility to partake in the study: (1) being of age 18 or older Finnish speaker who reports sleeping difficulties, (2) has (a) clinical insomnia or (b) subthreshold insomnia, i.e., received a score of 8 or higher on the Insomnia Severity Index (ISI^58^) but may not necessarily meet the full clinical criteria for insomnia disorder, (3) having access to Internet/email and telephone, (4) no lengthy breaks during the intervention period, and (5) not currently receiving other forms of psychological treatment for sleep complaints.

**Fig. 1:**
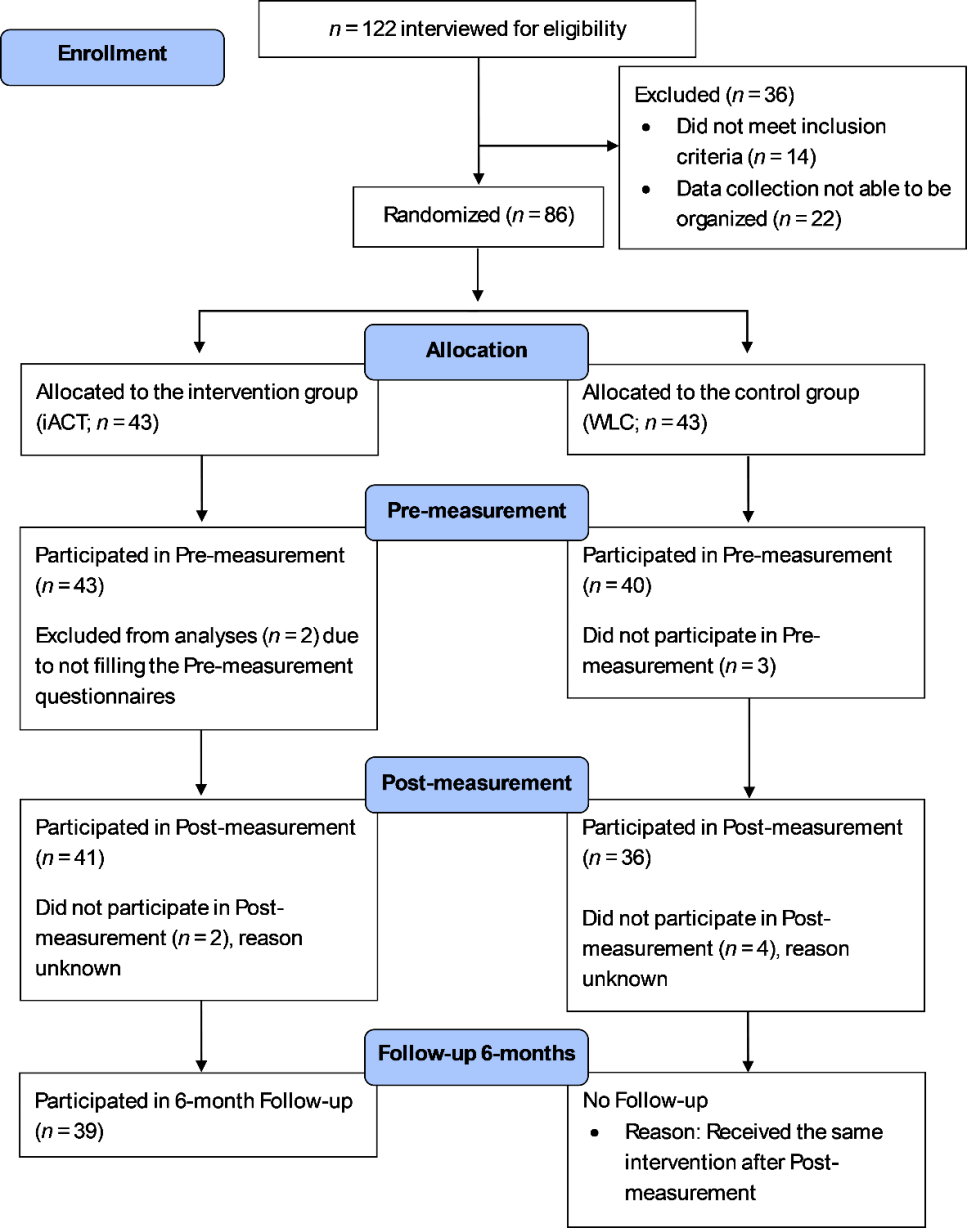
Participant flow chart.

Initially, 122 individuals contacted and were screened through a structured telephone interview conducted by two research assistants. Out of those contacting the clinic, 14 persons were excluded due to not fulfilling the eligibility criteria, and 22 otherwise eligible persons were excluded due to difficulties in organizing the measurements for them (due to resources and lack of research personnel). A total of 86 participants were thus included in the present study. See Fig. 1 for more detailed information on the flow of participants.

First, an independent person not involved in the study randomly assigned the included participants to either a group that received the online ACT intervention (iACT, *n* = 43) or a waitlist control group (WLC, *n* = 43) who would not receive treatment during the intervention period. Randomization was done using a simple randomization method and a randomizing tool (https://www.random.org/lists/).

After randomization, all participants received pre-measurement packages by mail, including information about the study, an informed consent form, a demographic information questionnaire including questions on medication and received treatment for sleeping problems, and questionnaires for subjective sleep complaints, symptoms, and psychological processes (see Measures section). A total of 83 participants (96.5%) returned the pre-measurement packages; however, two participants from the iACT group had not filled out the required pre-measurement questionnaires and were thus excluded (see Table 1 for the demographic characteristics and ISI scores of participants at pre-measurement). Nearly half of the participants (44%) had current sleep medication, and around a third (34%) had received earlier treatment for sleeping problems. Majority of the participants (70%) reported moderate or severe insomnia before the intervention.

**Table 1:** Participant demographic characteristics and ISI scores at pre-measurement.

Characteristics			iACT (*n* = 41)	WLC (*n* = 40)	All (*n* = 81)
Age	*M* (*SD*)		56.05 (10.94)	50.78 (15.26)	53.44 (13.43)
	Range		24–73	22–79	22–79
Gender *n* (%)	Female		32 (78%)	21 (52.5%)	53 (65.4%)
	Male		9 (22%)	19 (47.5%)	28 (34.6%)
Education^a^ *n* (%)	High		24 (58.5%)	24 (55.8%)	48 (59.3%)
	Middle		16 (39%)	14 (32.5%)	30 (37%)
	Low		1 (2.4%)	2 (4.7%)	3 (3.7%)
Marital status *n* (%)	Married or living together		36 (87.8%)	32 (74.4%)	68 (84%)
	Single or divorced		5 (12.2%)	8 (18.6%)	13 (16%)
Employment *n* (%)	Employed		19 (46.3%)	21 (48.8%)	40 (49.4%)
	Unemployed		2 (4.9%)	3 (7.0%)	5 (6.2%)
	Retired		15 (36.6%)	12 (27.9%)	27 (33.3%)
	Other^b^		5 (12.2%)	4 (9.3%)	9 (11.1%)
Current sleep medication *n* (%)		Yes	20 (48.8%)	16 (40%)	36 (44.4%)
		No	21 (51.2%)	24 (60%)	45 (55.6%)
Other current medication *n* (%)		Yes	27 (65.9%)	26 (65%)	53 (65.4%)
		No	14 (34.1%)	14 (35%)	28 (34.6%)
Earlier treatment for sleep *n* (%)		Yes	14 (34.1%)	14 (35%)	28 (34.6%)
		No	27 (65.9%)	26 (65%)	53 (65.4%)
Current psychological treatment *n* (%)		Yes	1 (2.4%)	3 (7.5%)	4 (4.9%)
		No	40 (97.6%)	37 (92.5%)	77 (95.1%)
Earlier psychological treatment *n* (%)		Yes	10 (24.4%)	11 (27.5%)	21 (25.9%)
		No	31 (75.6%)	29 (72.5%)	60 (74.1%)
ISI^c^ total score *M* (SD)			17.46 (3.87)	16.13 (4.06)	16.80 (4.00)
Subthreshold insomnia *n* (%)			9 (22%)	15 (37.5%)	24 (29.6%)
Moderate insomnia *n* (%)			25 (61%)	20 (50%)	45 (55.6%)
Severe insomnia *n* (%)			7 (17%)	5 (12.5%)	12 (14.8%)

Note. *M* = mean, *SD* = standard deviation.

^a^High = more than 12 years; middle = 9 to 12 years; low = less than 9 years.

^b^Includes students, those on sick leave, and homemakers.

^c^Insomnia Severity Index. A score of 7 or less indicates the absence of insomnia, 8–14 subthreshold insomnia, 15–21 moderate insomnia, and 22–28 severe insomnia^58^. A score of 8 or higher was required to be included in the present study.

Next, after the researchers received the pre-measurement packages, participants in the iACT group were granted access to the self-guided six-week-long online ACT intervention program by giving them a URL, a username, and a password. The WLC group was a no-treatment group, thus they did not have access to the intervention at this point.

Seven weeks later, a post-measurement package including questionnaires for subjective sleep complaints, symptoms, and psychological processes was sent by mail to all participants. Participants in the iACT group were instructed to fill out the questionnaires and bring them to the final in-person interview that was held after the intervention period. The interview’s purpose was to gather information about participants’ intervention experiences. After this, participants in the WLC group were granted access to the same online intervention program that iACT participants had received. Lastly, after six months, only the iACT group participants were contacted for follow-up. No follow-up data was gathered from the WLC group due to receiving the intervention after post-measurement (see also the effectiveness study by Lappalainen et al.^54^).

### Intervention

The online intervention program has been developed by the research team at the University of Jyväskylä. The program aims to help with sleeping problems by enhancing mindfulness and acceptance skills, psychological flexibility, and commitment towards value-based actions. The program contained a short page of information that provided instructions for sleep hygiene (e.g., maintaining a regular sleep/wake schedule^59^), stimulus control (e.g., leaving the bed/bedroom when awake for around 15 min^59^), and ACT-based instructions (e.g., take a break during the day to practice mindfulness). Each intervention week or module had its ACT-based theme with text, experiential audio exercises, and video clips (see Table 2).

**Table 2:** An overview of the intervention program’s modules.

Week & related ACT process	Module name and theme	Exercises
1) Values and value-based actions	Introduction *Broaden your horizon*: Identifying what matters the most in your life and acting accordingly.	Video clip: Values Video clip: Wise actions (value-based actions) Audio: Mindfulness of breathing
2) Mindfulness	*Present moment*: Noticing internal and external experiences. Noticing the movement of your breath. Living in the present moment.	Video clip: Mindfulness Audio: Mindful breathing, Mindful listening, Mindful sitting
3) Cognitive defusion	*Cognitive defusion*: The power of thoughts. Identifying thoughts and emotions and learning to describe, name, and welcome them.	Video clip: Wise mind Audio: Observing the stream of thoughts, Labeling thoughts
4) Self-as-context	*The observer’s stance*: Taking perspective, learning to view your thoughts and emotions from an observer’s stance.	Video clip: Observing Audio: Observer exercise
5) Acceptance	*Toward an attitude of acceptance*: Letting go of your struggle and learning acceptance towards your wakefulness and unwanted thoughts.	Video clip: Acceptance Audio: Scanner, A stone on the beach, An uninvited guest
6) Summary	*Summary of the program*: What have you learned? Plans for the future.	Audio: Body meditation

The first module addressed values and value-based actions, that is, how to move from struggling with insomnia towards what is meaningful in life and what actions align with these. The second addressed mindfulness and how to observe unwanted thoughts and emotions without judging them. The third module introduced the ability to create distance between oneself and thoughts and emotions and observe them as what they are. The fourth module introduced self-as-context and taking an observer’s role towards internal and external events. The fifth module addressed acceptance and how to create a more accepting attitude towards distressing thoughts. The final module summarized the whole intervention program. The program content was the same for all users, meaning that it did not contain tailored elements.

The participants were instructed to complete the module and its related exercises during their designated week before moving on to the next module. Data relating to the users or intervention program usage activity was not tracked within the program. Instead, the participants received two email reminders per week regarding the intervention program: The first message was sent to notify participants that they could now access the new module, and the second message was sent to motivate users to continue implementing the learned strategies in their lives. No other support or reminders were provided to the participants during the intervention.

### Measures

The Insomnia Severity Index (ISI^58,60^) was used in the present study as a screening tool and an indicator of symptom severity. The ISI has seven items (e.g., “How satisfied/dissatisfied are you with your current sleep pattern?”) that are answered on a scale from 0 to 4 to assess the perceived severity of insomnia symptoms, distress, and daytime impairment (Cronbach’s α = 0.90 – 0.91^58^). The total score ranges from 0 to 28, where an insomnia score of 7 or less indicates the absence of insomnia, 8–14 indicates subthreshold insomnia, 15–21 indicates moderate insomnia, and 22–28 indicates severe insomnia^58^. A total ISI score of 8 or higher was required to be included in the present study.

#### Main outcome measure

**Subjective Sleep Complaints**. The Basic Nordic Sleep Questionnaire (BNSQ^56^) was used as the main outcome measure to assess sleep quality, sleep duration during the night, sleep and waking time, daytime sleepiness, and snoring. Some of the questionnaire’s 25 items (e.g., “Have you had difficulties falling asleep during the past three months?”) are assessed on a five-point Likert scale from 1 to 5 (1 = never or less than once per month; 5 = every night or almost every night) and some require other input (e.g., “I sleep about __ hours per night”). A higher total sum score indicates poorer quality of sleep, higher fatigue, or more severe snoring and/or interrupted breathing.

#### Secondary outcome measures

**Daytime Sleepiness and Beliefs about Sleep**. The Epworth Sleepiness Scale (ESS^61^) consists of eight items that measure the level of experienced sleepiness during daytime. The questionnaire has been demonstrated to be a valid tool for evaluating the level of daytime sleepiness^62^. Each item has four response options estimating how the individual inadvertently dozes off when engaged in activities involving low levels of stimulation (0 = would never doze; 3 = high chance of dozing). The total score ranges from 0 to 24, where a score of 16 or higher indicates a high level of daytime sleepiness^61^.

The Dysfunctional Beliefs and Attitudes about Sleep Scale (DBAS^63^) assesses cognitions related to sleep and insomnia. The brief version of the questionnaire consists of 16 items that are answered on a Likert scale from 0 to 10 (e.g., “When I sleep poorly on one night, I know it will disturb my sleep schedule for the whole week”, 0 = strongly disagree; 10 = strongly agree). The brief version has demonstrated good internal consistency (Cronbach’s α = 0.77 – 0.79^63^). The total score can range from 0 to 160, where a higher score indicates a greater number of dysfunctional beliefs and attitudes about sleep. According to Morin et al.^63^, the dysfunctional nature of the questionnaire’s items stems from the level to which a person endorses them. That is, if a person heavily endorses a certain statement, it may lead to excessive worrying over it and thus contribute to sleeping problems.

**Psychological Processes**. The Five Facet Mindfulness Questionnaire (FFMQ^64^) was used as an indicator of mindfulness. The FFMQ is a self-report inventory for the assessment of mindfulness skills and consists of five facets: observing, describing, acting with awareness, non-judging, and non-reactivity (Cronbach’s α = 0.86 – 0.93 across facets^65^). The instrument includes 39 statements that are assessed on a five-point Likert scale (e.g., “When I’m walking, I deliberately notice the sensations of my body moving”, 1 = never or very rarely true; 5 = very often or always true). The item scores are added together (ranging from 39 to 195) where a higher score indicates a higher level of mindfulness skills.

The White Bear Suppression Inventory (WBSI^66,67^) was used to assess thought suppression. The instrument consists of 15 items that are answered on a five-point Likert scale (e.g., “There are things that I try not to think about”, 1 = strongly disagree; 5 = strongly agree). The total score is formed by adding all item answers together, thus ranging from 15 to 75 (Cronbach’s α = 0.87 – 0.89^66^). A higher score indicates a greater tendency to suppress thoughts.

**Psychological Symptoms**. The Symptom Checklist 90 (SCL-90^68,69^), contains 90 items that are answered on a five-point Likert scale (0 = none; 4 = extremely) to assess currently experienced psychological and physiological symptoms. The SCL-90 has several symptom dimensions and global indices, from which the Global Severity Index (GSI; Cronbach’s α = 0.97^70^) indicates the level of overall psychological distress. The GSI combines information concerning the number of symptoms and the depth of perceived distress^69^. The index is calculated as the mean score across all items, where a higher score indicates a higher level of distress.

The Beck Depression Inventory (BDI-II^71^) was used to assess depressive symptoms. The instrument contains 21 items with statements regarding depressive symptoms and their severity. The scale ranges from 0 to 63, where scores of 0–13 indicate no or few depressive symptoms, 14–19 mild depression, 20–28 moderate depression, and 29–63 severe depression. The BDI-II has been shown to have high internal consistency (Cronbach’s α = 0.86 – 0.92^72^) and high validity in evaluating depressive symptoms^73^.

### Statistical analysis

One demographic difference was discovered at baseline between the iACT group and the WLC group. The iACT group had significantly more female participants compared to male participants (females 76.2%, males 23.8%; *χ*^2^(1) = 4.80, *p* = .03), whereas the WLC group had a more equal representation (females 53.5%, males 46.5%). No other significant differences based on demographics were detected between the groups. The iACT group and the WLC group showed no differences at baseline in terms of sleep complaints (i.e., ISI, BNSQ), daytime sleepiness, and beliefs about sleep (i.e., ESS, DBAS), psychological processes (i.e., FFMQ, WBSI), or psychological symptoms (i.e., SCL-90, BDI-II).

First, the relationship of changes in sleep complaints with changes in daytime sleepiness, dysfunctional beliefs, and psychological processes and symptoms were examined with correlations. Next, mediation analyses were conducted to examine the effects of the online ACT intervention on sleep complaints through changes in daytime sleepiness, dysfunctional beliefs about sleep, and psychological processes and symptoms. We used the Mplus software version 8^74^ with maximum likelihood (ML) as the estimator. Significance was assessed with 1000 bootstrap resamples and 95% confidence intervals (95% CI). A total of 75 participants (iACT *n* = 39, WLC *n* = 36) had completed both pre- and post-measurements (i.e., enabling calculations of change scores) and were thus included in the analyses.

**Fig. 2:**
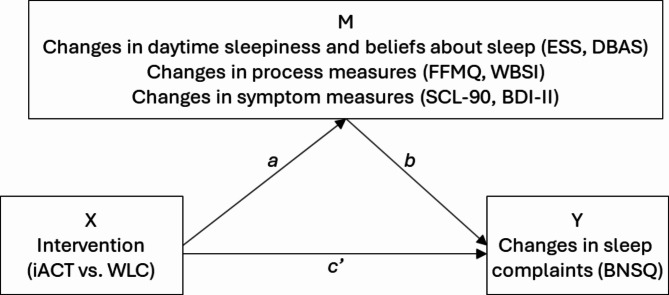
Mediation model for the present study.

We examined separate single-mediator models (Fig. 2), where the online ACT intervention (groups coded as 1 = iACT, 2 = WLC) was set as the independent variable (X) and change scores in sleep complaints (i.e., BNSQ) as the dependent variable (Y). Investigated mediators (M) were change scores in daytime sleepiness (i.e., ESS), dysfunctional beliefs about sleep (i.e., DBAS), psychological processes (i.e., FFMQ, WBSI), and psychological symptoms (i.e., SCL-90, BDI-II). In mediation, path *a* represents the relation of X to M, *b* represents the relation of M to Y, and *c’*represents the relation of X to Y adjusted for M^75^. The indirect effect is expressed by *a* × *b*, which is considered significant if the confidence interval does not include zero.

## Results

Table 3 presents the observed means and standard deviations for all measures at pre- and post-measurement and change scores between pre- and post-measurement.

**Table 3:** The observed means (*M*) and standard deviations (*SD*) for pre- and post-measurement and the changes between pre- and post-measurement. The change score mean represents a mean across calculated differences between pre- and post-measurement.

			iACT M (SD)	WLC M (SD)	All M (SD)
Outcome measure	BNSQ	Pre	21.68 (4.58)	21.65 (3.98)	21.67 (4.27)
		Post	19.79 (5.37)	21.38 (4.15)	20.56 (4.86)
		Change^a^	1.95 (2.78)	0.00 (3.40)	1.01 (3.22)
Sleepiness and beliefs	ESS	Pre	6.98 (4.64)	7.60 (4.17)	7.29 (4.40)
		Post	5.67 (3.84)	7.11 (4.76)	6.36 (4.34)
		Change^a^	1.24 (2.94)	0.28 (2.96)	0.77 (2.97)
	DBAS	Pre	96.39 (23.64)	87.93 (20.13)	92.21 (22.25)
		Post	86.10 (26.66)	87.39 (17.31)	86.72 (22.52)
		Change^a^	10.13 (18.02)	−3.14 (15.69)	3.76 (18.10)
Process measures	FFMQ	Pre	135.17 (15.87)	131.23 (18.49)	133.22 (17.22)
		Post	137.97 (17.70)	133.72 (18.81)	135.93 (18.24)
		Change^b^	2.62 (13.42)	0.56 (10.71)	1.63 (12.16)
	WBSI	Pre	44.46 (12.12)	44.85 (13.36)	44.65 (12.67)
		Post	39.33 (12.86)	42.17 (12.82)	40.69 (12.83)
		Change^c^	5.31 (8.60)	0.69 (8.44)	3.09 (8.78)
Symptom measures	SCL-90	Pre	0.57 (0.32)	0.65 (0.51)	0.61 (0.43)
		Post	0.38 (0.33)	0.51 (0.35)	0.44 (0.34)
		Change^d^	0.20 (0.35)	0.05 (0.22)	0.13 (0.30)
	BDI-II	Pre	12.20 (6.73)	12.38 (9.22)	12.28 (8.01)
		Post	7.69 (6.49)	10.39 (7.05)	8.99 (6.85)
		Change^d^	4.67 (5.26)	0.33 (3.36)	2.59 (4.93)

Note. BNSQ = Basic Nordic Sleep Questionnaire. ESS = Epworth Sleepiness Scale. DBAS = Dysfunctional Beliefs and Attitudes about Sleep Scale. FFMQ = Five Facet Mindfulness Questionnaire. WBSI = White Bear Suppression Inventory. SCL-90 = Symptom Checklist 90. BDI-II = Beck Depression Inventory.

^a^A positive change score indicates a decrease in sleep complaints, daytime sleepiness, and dysfunctional beliefs and a negative score indicates an increase.

^b^A positive change score indicates an increase in mindfulness skills and a negative score indicates a decrease.

^c^A positive change score indicates a decrease in thought suppression and a negative score indicates an increase.

^d^A positive change score indicates a decrease in symptoms and a negative score indicates an increase.

Changes in sleep complaints correlated significantly with changes in daytime sleepiness (*r* = .24, *p* = .04), changes in thought suppression (*r* = .47, *p* ≤ .001), and changes in depressive symptoms (*r* = .29, *p* = .01). That is, improvement in subjective sleep complaints were associated with decreased levels of daytime sleepiness, thought suppression, and depressive symptoms. In turn, changes in dysfunctional beliefs, mindfulness, or psychological distress were not found to correlate with changes in subjective sleep complaints (*p* > .05 for all).

The total effect (i.e., the *c* coefficient that includes indirect and direct effects) was significant (*p* = .006, SE = 0.11, 95% CI [−0.50, −0.07]), meaning that taking part in the online ACT intervention alleviated participants’ subjective sleep complaints. Two significant mediators were identified in the models (see Table 4).

**Table 4:** Standardized *p*-values (standard errors in parentheses) and 95% confidence intervals for single-mediator models. Groups coded as 1 = iACT, 2 = WLC.

Mediator	Path				a × b 95% CI	
	*a*	*b*	*c’*	*a* × *b*	Lower	Upper
Sleepiness and beliefs						
ESS	0.16	0.12	0.02	0.37	−0.15	0.01
	(0.12)	(0.13)	(0.12)	(0.04)		
DBAS	≤ 0.001	0.42	0.02	0.46	−1.04	0.27
	(0.10)***	(0.12)	(0.12)*	(0.05)		
Process measures						
FFMQ	0.47	0.50	0.01	0.74	−0.08	0.01
	(0.12)	(0.11)	(0.11)**	(0.02)		
WBSI	0.01	≤ 0.001	0.08	0.02	−0.21	−0.03
	(0.10)**	(0.09)***	(0.11)	(0.05)*		
Symptom measures						
SCL-90	0.04	0.59	0.01	0.66	−0.09	0.03
	(0.12)*	(0.09)	(0.12)**	(0.03)		
BDI-II	≤ 0.001	0.03	0.07	0.05	−0.18	−0.01
	(0.09)***	(0.09)*	(0.12)	(0.04)*		

**Fig. 3:**
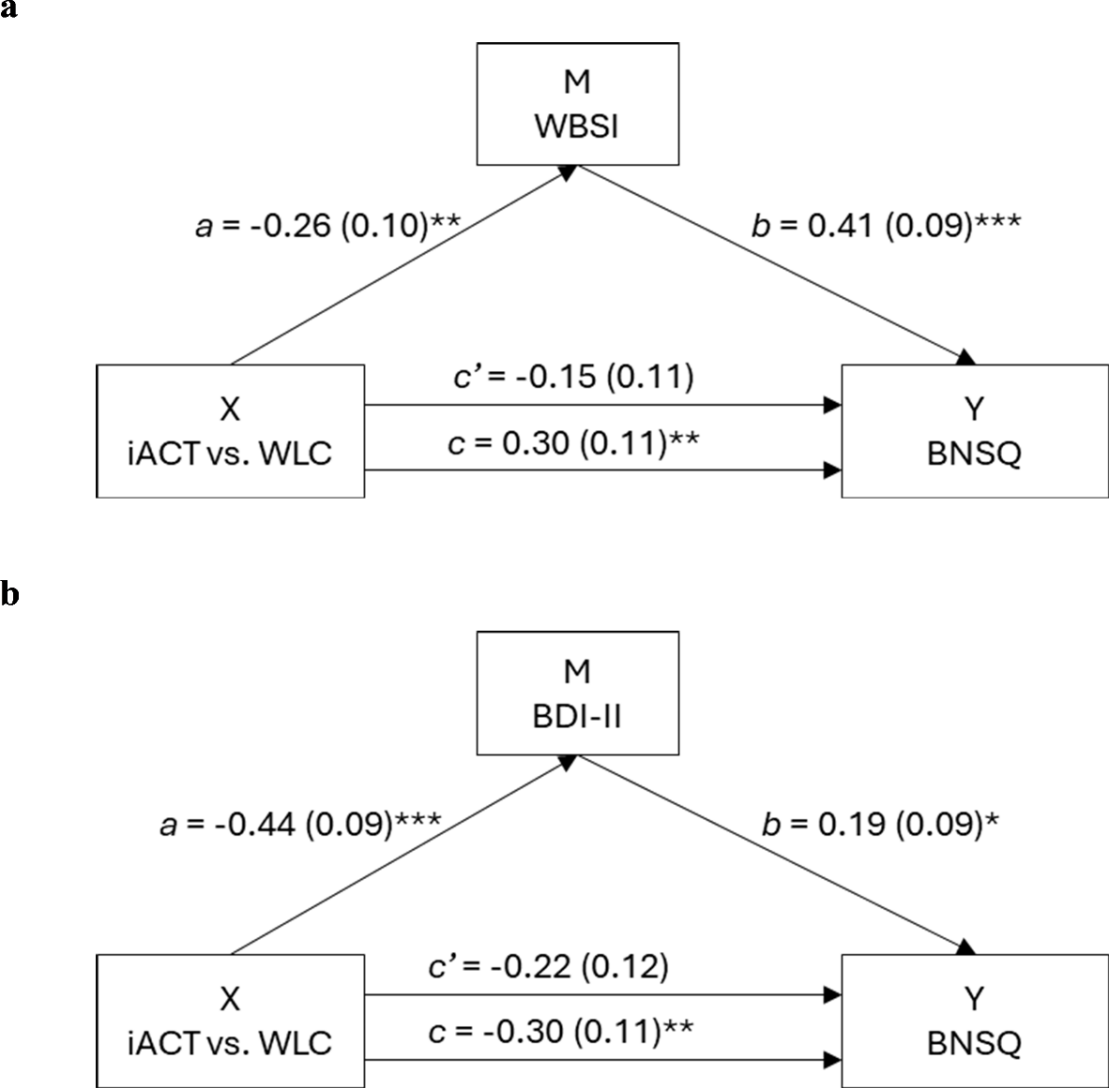
Illustrations for significant mediation analysis results with estimates, standard errors in parentheses, and *p*-value. *Note.* Standardized mediation results for the models where changes in (**a**) thought suppression (WBSI) and (**b**) depressive symptoms (BDI-II) mediated the effects of online ACT (iACT, WLC = waitlist control) intervention to changes in sleep complaints (BNSQ). Path *c* represents the total effect of independent variable X to dependent variable Y, path *a* represents the effect of X to the mediator M, path *b* represents the effect of M to Y, and path *c’* represents the effect of X to Y adjusted for M. * *p* ≤ .05; ** *p* ≤ .01; *** *p* ≤ .001.

First, changes in thought suppression were found to mediate the effect of online ACT to changes in subjective sleep complaints (see Fig. 3, illustration a). The indirect effect remained significant when comparing the results with unstandardized results (*p* = .03, *SE* = 0.32, 95% CI [−1.47, −0.20]). Second, changes in depressive symptoms were also found to mediate the effect of online ACT to sleep complaints (see Fig. 3 illustration b), with the effect approaching the threshold for significance in the unstandardized results (*p* = .06, *SE* = 0.29, 95% CI [−1.21, −0.06]). In both models, the *c’*coefficient was not significant, which refers to full mediation^75^.

Daytime sleepiness, dysfunctional beliefs about sleep, mindfulness, and overall psychological distress were not found to mediate the effect of online ACT on sleep complaints (i.e., 95% CI for *a* × *b* included zero). Overall, the results suggested that the online ACT intervention decreased participants’ subjective sleep complaints via decreases in thought suppression and depressive symptoms.

## Discussion

This study examined the indirect effects of a six-week online ACT intervention for adults experiencing clinical (i.e., participants with an ISI score of 15 or higher) or sub-clinical insomnia (i.e., participants with an ISI score of 8–14). Daytime sleepiness, beliefs about sleep, mindfulness, thought suppression, overall psychological distress, and depressive symptoms were investigated as possible mediators. We found that the online ACT intervention decreased thought suppression and depressive symptoms, which, in turn, decreased subjective sleep complaints.

Experiential avoidance, i.e., unwillingness to be in contact with private experiences, has been underlined as a key construct in psychopathology^40,41^. Previously, a decrease in experiential avoidance during a group-based ACT intervention was found to associate with better sleep quality, acceptance of sleep problems, lower dysfunctional beliefs about sleep, and lower difficulties in emotion regulation^55^. Thought suppression represents a form of experiential avoidance and was measured in the present study by the WBSI questionnaire (consisting of items such as “There are things that I try not to think about” and “I have thoughts that I cannot stop”). We found that an occurring decrease in thought suppression mediated the effects of an online ACT intervention to adults’ subjective sleep complaints. This falls in line with other studies that have investigated thought suppression as a mediator in relation to other health concerns such as post-traumatic stress and substance cravings^51–53^. Thought suppression has been noted to be a typical cognitive strategy for individuals who struggle with insomnia^48^.

From an ACT perspective, the present results could indicate that an intervention that targets insomnia may need to focus on lowering avoidance-related processes and increase acceptance as the corresponding flexibility process. Acceptance has been underlined as a key component of ACT and to predict well-being, functioning, and quality of life^21,36^. Acceptance and mindfulness have been suggested to predict improvements in sleep problems^17,37^. However, before drawing firm conclusions, further research is required to investigate whether acceptance has a unique role in mediating intervention effects compared to other flexibility processes. In this, for example, the Sleep Problem Acceptance Questionnaire (SPAQ^76^) could be utilized to provide further insights. In addition, the present study did not find support for changes in mindfulness being a mediator for the effects of online ACT on sleep complaints. One explanation for this could be, as Lappalainen et al.^54^ suggested, that the participants’ psychological flexibility and mindfulness skills were already relatively high before the online ACT intervention, which leaves less room for skill improvement during the intervention. Therefore, future research is encouraged to be conducted regarding the more specific roles of mindfulness, acceptance, and experiential avoidance as possible mechanisms of online ACT in treating insomnia.

Dysfunctional beliefs about sleep have been found to mediate the effects of online CBT-I^8^, but the present study did not identify similar mechanisms concerning online ACT. The difference in the aims of CBT and ACT could explain the difference in results: CBT focuses on changing cognitive structures and ACT focuses on functions, acceptance, and defusion. It is possible that people’s beliefs about sleep may change during an ACT intervention (e.g., Lappalainen et al.^54^), but ACT does not per se target cognitions in a similar manner like CBT does. The DBAS as an instrument is useful in identifying dysfunctional cognitions related to sleep^57^, but does not explore their functions^21^.

Previously, it has been suggested that decreases in sleep complaints may lead to decreases in other symptoms such as depression and anxiety^77^. The present results indicated that changes in depressive symptoms (measured with BDI-II) mediated the effects of online ACT on subjective sleep complaints. Depressive symptoms are connected to thought suppression and psychological inflexibility^78^. Depressed individuals often engage in the suppression of distressing thoughts in an attempt to achieve a positive mood^79^. However, important to note is that the present study found stronger evidence for the role of thought suppression as a mediator, compared to the more suggestive results concerning depressive symptoms. In addition, overall psychological distress (measured with SCL-90) was not found to mediate the effect. It seems possible that a global measure for distress may not accurately reflect associations of mood-related symptoms to psychological inflexibility and insomnia. It could also be that depressive symptoms have a unique connection with thought suppression in the context of insomnia, which were not accurately reflected in the models examined in the present study and would thus require further research.

### Limitations and recommendations for future studies

Some considerations should be addressed before attempting to generalize the present study’s results. First, the ISI questionnaire was used as a screening tool in participant recruitment process but could possibly serve as well as an outcome measure. The ISI questionnaire is commonly used as a brief screening tool in clinical settings^57^, and is more specific to insomnia-related problems compared to the outcome measure used in the present study. The BNSQ was chosen in the present study as the outcome measure for its wider approach to sleep complaints. However, it is possible that utilizing instruments more specific to insomnia could reveal valuable additional information in relation to underlying mechanisms of online ACT.

Second, the present study did not control for primary or comorbid insomnia. On the one hand, this reflects “the real world”, where sleep complaints rarely occur without other conditions^80^. On the other hand, this could affect the results’ generalizability. Given that around a third of the participants in the present study showed at least mild depressive symptoms (i.e., a BDI-II scores of 14 or higher) before the intervention, it is possible that the sample consisted of participants with both primary and comorbid insomnia. In future research, primary and comorbid insomnia could be controlled for in more detail, which could offer important additional information on mechanisms of change as well as the directions of causality.

Third, adherence was not controlled for in the present study. A separate adherence criterion was not established in the present study, rather, the criterion for participants to be included in this study’s analyses was that they had returned the pre- and post-measurement questionnaire packages. Adhering, or, receiving a sufficient “dosage” of an intervention is important in terms of outcomes^81^. Therefore, utilizing different objective (e.g., login information, total time of program usage) or subjective (e.g., asking about the extent of usage) measures for assessing adherence could be used to obtain further information on engagement, individual differences, and responses to intervention.

Fourth, although information on current sleep medication was gathered and considered in randomization (i.e., no baseline differences existed between groups in terms of having vs. not having current medication), the present study did not control for the possible effects of actual intake of sleep medication during intervention. It is possible that some of the participants with current sleep medication may have a more regular usage while others take it only occasionally. More accurate information would be needed regarding participants’ sleep medication to draw firmer conclusions on its possible effects on sleep during intervention.

Lastly, since the WLC group received the intervention after post-measurement, there is no follow-up data available regarding the control group. As the control group was a waitlist control, this limits our ability to make firm conclusions on whether the observed mechanisms of change are unique to online ACT. Therefore, future studies could be conducted with comparisons of different interventions for insomnia, in addition to inspecting intervention effects with longer follow-up periods^21,22^.

## Conclusions

The possibilities of utilizing ACT in treating insomnia has been the focus of small but growing research literature, which has lately experienced an extension to also online-based ACT. Overall, ACT-based interventions, in online and face-to-face form, have been demonstrated to be beneficial options for treating insomnia in adults. This study examined the mediators in a six-week online ACT intervention for adults suffering from subclinical and clinical insomnia. The results showed that the online ACT intervention decreased participants’ thought suppression (representing a form of experiential avoidance) and depressive symptoms, which were associated with decreased subjective sleep complaints. We suggest that an ACT intervention that targets insomnia may be beneficial through its focus on lowering avoidance of unwanted experiences and increasing acceptance of their presence, although future research with more process-specific measures is encouraged before drawing firm conclusions. Further investigations on processes such as acceptance or experiential avoidance seem of interest in relation to utilizing ACT for adults with insomnia symptoms. The results obtained in the present study lay a foundation for future studies by encouraging the utilization of more population-specific approaches and refined instruments to investigate the other possible underlying mechanisms in online ACT for insomnia.

## Data Availability

The datasets generated and/or analyzed during the current study are not publicly available but are available from the corresponding author on a reasonable request.
